# Selective pertraction of dicarboxylic acids from simulated *Rhizopus oryzae* fermentation broths

**DOI:** 10.1038/s41598-023-34100-3

**Published:** 2023-05-03

**Authors:** Lenuta Kloetzer, Alexandra Cristina Blaga, Dan Caşcaval, Anca Irina Galaction

**Affiliations:** 1 Department of Organic, Biochemical, and Food Engineering, “Cristofor Simionescu” Faculty of Chemical Engineering and Environmental Protection, “Gheorghe Asachi” Technical University of Iasi, Iasi, Romania; 2 Department of Biomedical Sciences, Faculty of Medical Bioengineering, “Grigore T. Popa” University of Medicine and Pharmacy of Iasi, Iasi, Romania

**Keywords:** Chemical engineering, Process chemistry

## Abstract

Fumaric, malic and succinic acids have been selectively separated by facilitated pertraction with Amberlite LA-2, using *n*-heptane as liquid membrane. The feed phase consisted on viscous aqueous solution with similar mixture of carboxylic acids and viscosity as those of *Rhizopus oryzae* fermentation broth. Due to the differences between the acidities and molecule size of these acids, it is possible to selectively recover fumaric acid from the initial solution. The pH-gradient between the feed and stripping phases, as well as carrier concentration in the liquid membrane represent the main process parameters influencing the pertraction selectivity. Among them, Amberlite LA-2 concentration exhibits the most important control on the selectivity factor S, the maximum value of S being reached for carrier concentration of 30 g/l. The increase of feed phase viscosity amplified the magnitude of these influences on pertraction selectivity, due to the hindrance of acids diffusion towards the region where their reaction with Amberlite LA-2 occurs, effect more important for malic acid. Therefore, by modifying the viscosity from 1 to 24 cP, the maximum value of selectivity factor was increased from 12 to 18.8.

## Introduction

Fumaric acid is one of the dicarboxylic acids with numerous applications in the food, pharmaceutical, chemical, and polymers industries^1,2^. For these reasons, the interest on this acid production at industrial scale was increased in the last years, fumaric acid being produced by chemical methods, from maleic anhydride, benzene, or *n*-butane, and by biochemical ones, using fermentation with fungus of *Rhizopus spp.* (mainly *Rhizopus oryzae* and *Rhizopus arrhizus*)^2–4^. By comparing the two technological methods on the basis of material and energy consumption, especially in the context of their cost’s fluctuation, as well as of the environment impact, the biochemical technologies are more attractive. Therefore, the fumaric acid production by biosynthesis using strains of *Rhizopus spp.* offers advantages in term of high yield, but also advantages related to the use of biocompatible raw materials (in this case substrates), and the absence of toxic by-products (generated in the case of chemical methods), which makes it an eco-friendly process^3,4^.

The final broth of *Rhizopus spp.* fermentation contains a mixture of organic acids, with fumaric acid as the main product. This mixture includes also smaller amounts of few secondary organic acids, the most important being malic and succinic ones^1–3^. Generally, the percentage composition of this carboxylic acids’ mixture is: 85–90% fumaric acid, 7–9% malic acid, and 2–7% succinic acid^5–7^. Although the biochemical method is attractive from the above-mentioned reasons, the industrially applied methods for separating selectively the biosynthetic acids from *Rhizopus spp.* broths, namely electrodialysis, crystallization in acidic media, ion exchange, and precipitation as calcium salt, raise some problems concerning the generation of waste by-products with potential negative impact on the environment (important amount of calcium sulphate sludge, acidic wastewaters, etc.)^2,4^.

Generally, the liquid–liquid extraction represents an accessible and efficient alternative for many downstream processes at large-scale biotechnologies. However, its application for ionizable compounds, in this case carboxylic acids, is rather limited due to these compounds’ low solubility in the industrial organic solvents. Therefore, the extraction yield of fumaric acid in usual hydrophobic solvents is less than 10%, while the maximum extraction degrees for malic and succinic acids are around 30%, being reached for alcohols with aliphatic chains over 4 carbon atoms (30–37%)^8^. In these cases, the efficiency of liquid–liquid extraction can be significantly improved by adding an extractant of aminic type into the solvent phase^8^. The separation process is called reactive extraction, the extractant used for individual and selective removal of fumaric, malic, and succinic acids from their biosynthetic mixture being Amberlite LA-2^9,10^. According to the previous experiments, depending on the value of solvent dielectric constants, the reactive extraction with Amberlite LA-2 of these acids offers an increased efficiency of separation, their extraction yields becoming over 95%^10^.

Bulk pertraction, based on the transfer of a solute between two aqueous phases separated by an organic immiscible solvent layer, is a separation technique that combines extraction and re-extraction (stripping) in the same equipment^11^. It offers several advantages related to the use of solvent in a small quantity, since due to re-extraction it is continuously regenerated, but more important pertraction allows transferring a compound from a phase with lower concentration into one with higher concentration, as long as the driving force (pH difference or ionic straight between aqueous phases) is maintained^12^. In the organic phase (water immiscible solvent) several carriers (long chain amines, ionic liquids, organophosphorus compounds, crown ethers) can be dissolved that can improve pertraction performance. Its mechanism is influenced by the carriers and solute characteristics and concentration (that influences the extraction and re-extraction mechanism), but also by the pH difference between the two aqueous phases.

The previous studies confirmed that the reactive extraction with Amberlite LA-2 can be used for separating selectively the acids mixture obtained by *Rhizopus oryzae* fermentation^10^. As it was above presented, pertraction represents a development of the reactive extraction method, mainly due to its advantages and integrated steps. Moreover, the previous experiments have been carried out by separating the carboxylic acids from aqueous solutions with viscosity equal to that of water at considered temperature, not from viscous solutions similar to the real fermentation broths^10^.

In this context, this work investigates the possibility to use pertraction for selective separation of fumaric, malic, and succinic acids from aqueous solutions simulating the composition and viscosity of real fermentation broths. In this purpose, the facilitated pertraction with Amberlite LA-2, as carrier, dissolved into *n*-heptane, as liquid membrane, has been applied. The pertraction mechanism and selectivity have been analyzed in direct relation to the pH-gradient between the two aqueous phases, mixed acids solution (feed phase) viscosity, carrier concentration in the liquid membrane, and mixing intensity.

## Methods

**Figure 1:**
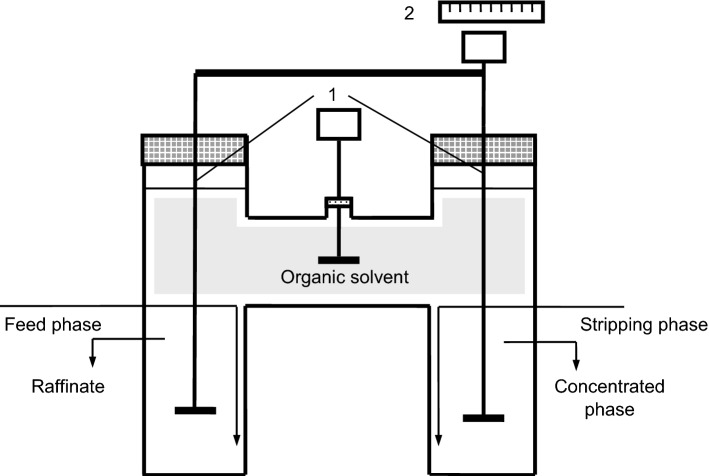
Pertraction cell and the general mechanism of pertraction (1—blade stirrers, 2—tachometer).

The experiments have been carried out using the pertraction equipment described in the previous papers (Fig. 1)^13^. The pertraction cell was designed and operated for obtaining and maintaining the solvent layer between the two aqueous phases without needing surfactants, only on the basis of the densities difference between the three phases. The pertraction cell consists on a reverse U-shaped glass pipe having an inner diameter of 45 mm and a total volume of 450 ml, the volume of each compartment being of 150 ml. The aqueous solutions and the solvent phase have been independently mixed by means of double blade stirrers with 6 mm diameter and 3 mm height, having a rotation speed of 300 rpm. The interfacial area of mass transfer, both for extraction and for re-extraction, was of 1.59 × 10^−3^ m^2^. The interfaces between the phases remained flat, and hence the interfacial area constant, for the considered rotation speed value.

The operating regime of the pertraction cell was the pseudosteady-state one, considering the steady-state regime related to the feed and stripping phases, and the unsteady-state mode related to the solvent phase. Each aqueous solution has been fed with a volumetric flow rate of 2.5 l/h, the pertraction temperature being maintained at 25 °C.

The solvent phase, the liquid membrane, consisted of *n*-heptane (dielectric constant of 1.90 at 25°C^14^) in which Amberlite LA-2, the carrier, has been dissolved. The carrier concentration varied between 5 and 300 g/l (0.014–0.85 M).

The feed phase simulated the final broths obtained by fermentation with *Rhizopus oryzae* in terms of viscosity and acids composition. Therefore, the feed phase consisted on aqueous solutions of carboxymethylcellulose sodium salt whose viscosity varied from that of pure water to that corresponding to *Rhizopus oryzae* broths, namely 24 cP^6^. The viscosity has been measured before and after each experiment by means of Viscotester 6 Plus equipment (Haake) at 25 °C.

The initial composition of the feed phase respected that obtained by fermentation: 87% (5 g/l–4.3 × 10^−2^ M) fumaric acid, 9% (0.5 g/l–3.7 × 10^−3^ M) malic acid, and 4% (0.25 g/l–2.1 × 10^−3^ M) succinic acid^6^. The pH-value of the feed phase has been adjusted and maintained between 1 and 7 with solution of 3% sulfuric acid or 3% sodium hydroxide, depending on the experimental program.

The stripping phase consisted in solutions of sodium hydroxide with pH = 7—12. The control of pH-values of feed and stripping phase was made by two digital pH-meters of Consort C836 type and has been recorded throughout each work. Any pH change was recorded during the pertraction experiments.

The pertraction process was analyzed based on the initial and final mass flows of the studied carboxylic acids, permeability, and selectivity factors. For calculating these parameters, the acids concentrations in the feed and stripping phases have been measured and the mass balance for the entire pertraction system has been used:

- Initial mass flow:
  1 $${\text{n}}_{{\text{i}}} = \frac{{{\text{W}} \cdot \left( {{\text{C}}_{{0}} - {\text{C}}_{{\text{R}}} } \right)}}{{\text{A}}},\;{\text{mol}}/{\text{m}}^{{2}} \;{\text{h}}$$
- Final mass flow:
  2 $${\text{n}}_{{\text{f}}} = \frac{{{\text{W}} \cdot {\text{C}}_{{\text{S}}} }}{{\text{A}}},\;{\text{mol}}/{\text{m}}^{{2}} \;{\text{h}}$$
- Permeability factor:
  3 $${\text{P}} = \frac{{{\text{n}}_{{\text{f}}} }}{{{\text{n}}_{{\text{i}}} }}, - .$$

Fumaric, malic, and succinic acids concentrations have been determined by high performance liquid chromatography technique (HPLC equipment of UltiMate 3000 Dionex type) with an AcclaimTM OA column (4 mm diameter, 150 mm length, 5 μm porous particle), provided with UV detector at 210 nm^10^. The mobile phase was a solution of 100 nM sodium sulfate, having the pH adjusted at value 2.65 with methanesulfonic acid. The flow rate of mobile phase was 0.6 ml/min. The analysis has been carried out at 30 °C^10^.

Each experiment included in the working program has been carried out for two or three times. The discussions took into account the average values of the above-mentioned parameters. The maximum experimental error was of 7.71%.

## Results and discussion

Generally, the efficiency of facilitated bulk pertraction is controlled by the pH difference between the initial and final aqueous phases, liquid membrane polarity, extractant concentration in liquid membrane, and phases mixing intensity. The pH-gradient represents one of the most important factors. It determines the solute ionization in the aqueous phases, thus controlling the extraction efficiency and the re-extraction process, as well as the acids rate of transport through the membrane phase.

**Figure 2:**
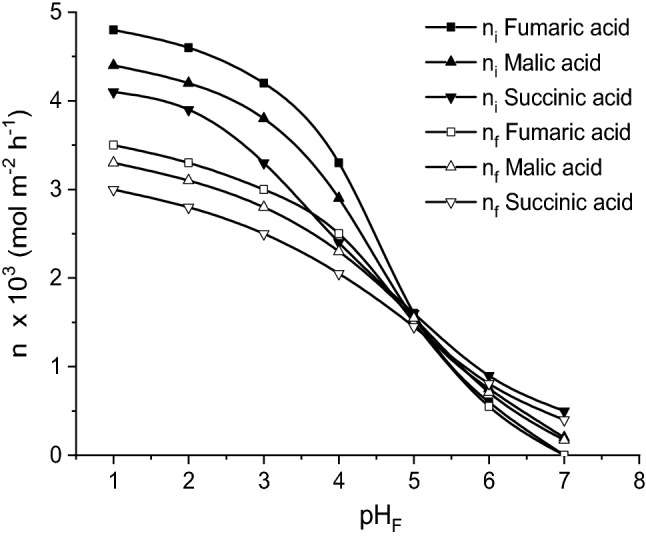
Influence of pH-value of feed phase on fumaric, malic, and succinic acids mass flows (carrier concentration = 200 g/l, pH of stripping phase = 10, feed phase viscosity = 1 cP).

Figure 2 indicates, for all dicarboxylic acids, the decrease of initial mass flows with the increase of pH-value of feed phase, pH_F_. This effect is the result of reactive extraction with Amberlite LA-2 performance lowering at the interface between the feed phase and liquid membrane due to the solutes partial dissociation, at one carboxylic group, process that is less important for pH_F_ values below 3. Although the dependence between the initial mass flow and pH_F_ is similar for all three acids, for pH_F_ < 5, the order of reduction of initial mass flow is fumaric acid > malic acid > succinic acid. This order of variation is correlated to the solutes acidity, because the acidity controls the rate of interfacial reaction between solute and carrier (at 25 °C, Ka_1_ = 9.33 × 10^−4^ for fumaric acid, Ka_1_ = 3.98 × 10^−4^ for malic acid, Ka_1_ = 6.92 × 10^−5^ for succinic acid^14^).

However, the rate of interfacial reaction between acids and carrier is also controlled by the structure of the interfacial compounds by means of the number of carrier molecules stoichiometrically needed. According to the previous results on dicarboxylic acids reactive extraction using *n*-heptane as solvent phase, the interfacial interactions could be of hydrogen bonding type with the un-dissociated carboxylic groups, or of ionic type, if the acid dissociates in the aqueous solution^9^:

$${\text{R}}\left( {{\text{COOH}}} \right)_{{{2}({\text{aq}})}} + {\text{mQ}}_{{({\text{o}})}} \rightleftarrows {\text{R}}\left( {{\text{COOH}}} \right)_{{2}} .{\text{Q}}_{{{\text{m}}({\text{o}})}}$$

Q represents Amberlite LA-2. Thus, the previous studies indicated that the structures of the interfacial compound are R(COOH)_2_.Q_2_ for fumaric and malic acids, while for succinic acid becomes R(COOH)_2_.Q_4_^9^. Consequently, for pH_F_ below 5, the order of the acids initial mass flows diminution is the result of the cumulated effects of the acidity decreasing from fumaric to succinic acid and of the increased complexity of interfacial acid—carrier compound structure, namely of the increased number of Amberlite LA-2 molecules required for reacting with the solute. Both effects lead to the intensification of the interfacial reaction rate from succinic to fumaric acid.

This order is reversed for pH_F_ > 5, the initial flow of succinic acid becoming the highest one, while that of fumaric acid the lowest (Fig. 2). In this case, the determinant role has been attributed to the different solute solubility with different acidity into a low-polar solvent. As it was reported in literature, the low-polar solvents can solubilize low- or non-polar molecules^8^. According to this result, by increasing the acidity of the solutes the ability of *n*-heptane to extract them is diminished, that leading to the following order of extraction efficiency: succinic acid > malic acid > fumaric acid.

The dependences between the acids final mass flows and pH-value of feed phase are similar with those recorded for their initial mass flows, in accordance to a direct relation to the acids transferred into the membrane layer (Fig. 2).

**Figure 3:**
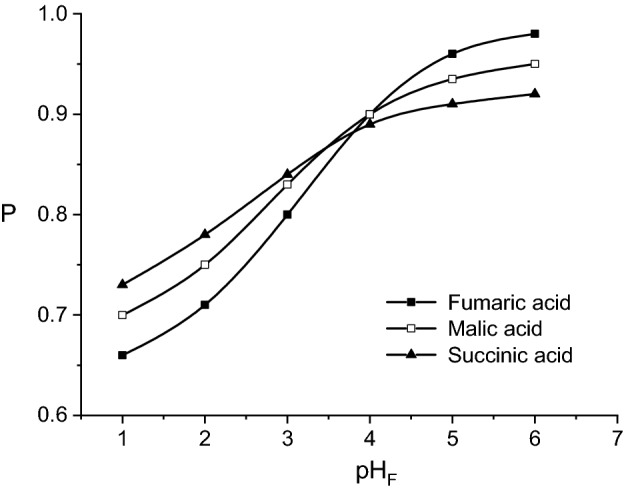
Influence of pH-value of feed phase on fumaric, malic, and succinic acids permeability factors (carrier concentration = 200 g/l, pH of stripping phase = 10, feed phase viscosity = 1 cP).

Contrary to the influence on mass flows, the increase of pH_F_ generates the increase of permeability factor for all three dicarboxylic acids, the initial mass flows becoming closer to the corresponding final ones by increasing the pH_F_-value towards the neutral values domain, on account of a small acid quantity transferred into the membrane phase (Fig. 3).

Moreover, Fig. 3 suggests two domains of permeability factors variation, below and over pH_F_ = 5. For pH_F_ < 5, the dependences indicate that the transport capacity of liquid membrane is affected by increasing the acidity of transferred solute, while for pH_F_ > 5 the acidity exhibits a positive effect.

The variations of permeability factors are controlled by the rate of interfacial reactions between acid and carrier, respectively between acid-carrier compound and sodium hydroxide. Generally, the increase of solute acidity, implicitly generating the strength increase of the bond between solute and carrier, and the increase of the solute-carrier compound complexity leads to the amplification of kinetic resistance to the re-extraction process. The relative importance of the two factors on the solute transfer from liquid membrane to the stripping phase could be changed by varying the value of pH_F_. For pH_F_ < 5, the corresponding order of the acids permeability factors is mainly the result of the acidity variation for dicarboxylic acids, contrary to the variation of the kinetic resistance for the re-extraction process, as it was above discussed. Therefore, the order of permeability factors is: succinic acid > malic acid > fumaric acid.

For pH_F_ domain over 5, the main factor controlling the membrane permeability becomes the complexity of the chemical structure of the acid—carrier compound in the membrane phase. In this case, the permeability factors are reduced from fumaric acid to succinic acid.

In both cases, the effects of the two mentioned factors can be cumulated with the higher initial mass flows of fumaric and malic acids from the feed phase to membrane one for pH_F_ below 5, or higher initial mass flow of succinic acid for pH_F_ over 5, these exhibiting a negative influence on the permeability.

The re-extraction of dicarboxylic acids from liquid membrane to the stripping phase is based on the following interfacial reaction with sodium hydroxide:

$${\text{R}}\left( {{\text{COOH}}} \right)_{{2}} .{\text{Q}}_{{{\text{m}}({\text{o}})}} + {\text{NaOH}}_{{({\text{aq}})}} \rightleftarrows {\text{R}}\left( {{\text{COO}}^{ - } {\text{Na}}^{ + } } \right)_{{{2}({\text{aq}})}} + {\text{mQ}}_{{({\text{o}})}}$$

**Figure 4:**
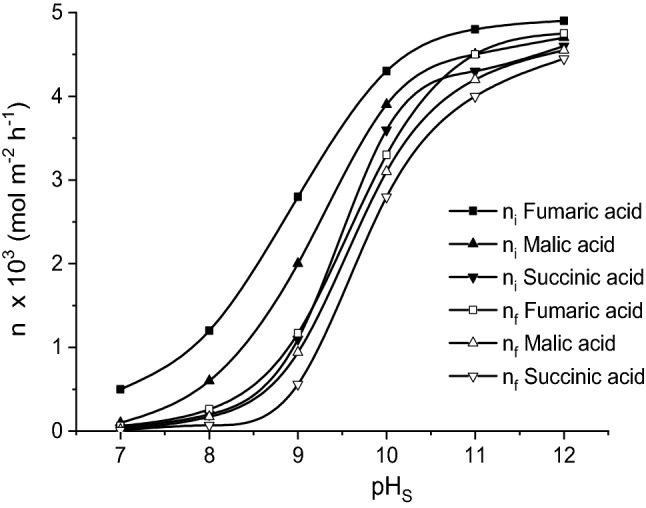
Influence of pH-value of stripping phase on fumaric, malic, and succinic acids mass flows (carrier concentration = 200 g/l, pH of feed phase = 2, feed phase viscosity = 1 cP).

According to this mechanism, the increase of final aqueous phase pH, pH_S_, exhibits a positive effect the final mass flows of the three acids (Fig. 4), accelerating the sodium salts formation rate, and, consequently, acids re-extraction in the stripping solution, this generating an important gradient concentration between the two aqueous phases, that lead to a higher initial mass flow. However, Fig. 3 suggests an important increase of mass flows in the pH_S_ domain 8–10. At pH_S_-values close to the neutral domain, the solutes fluxes from feed phase to liquid membrane are very low, while those from liquid membrane to stripping phase tend to 0. At higher pH_S_-values, both the initial and the final mass flows are maintained constant. The order of the mass flows for a given pH_S_-value is directly related to the order of the solutes acidities_._

Figure 4 indicates the continuous increase of the permeability factors for all considered acids with the increase of stripping phase pH-value. This dependence suggests that the positive effect of pH_S_ increase is relatively more important on final mass flows compared to the initial ones, as the result of the above discussed phenomena.

**Figure 5:**
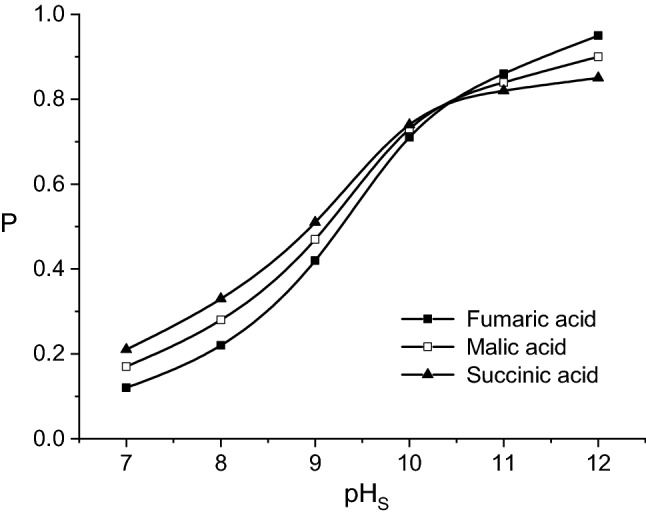
Influence of pH-value of stripping phase on fumaric, malic, and succinic acids permeability factors (carrier concentration = 200 g/l, pH of feed phase = 2, feed phase viscosity = 1 cP).

By comparing the relative magnitude of the effect of pH_S_ increase on the permeability factors, the strongest influence has been recorded for fumaric acid. Therefore, the increasing order of the permeability factors at pH_S_ = 7 was fumaric acid < malic acid < succinic acid, being completely reversed at pH_S_ = 12, namely succinic acid < malic acid < fumaric acid (Fig. 5). The turning point corresponded to pH_S_ = 10.

At pH-values of stripping phase close to neutral domain, the sequence of the permeability factors increase is the result of kinetic resistance amplification in the re-extraction process from succinic acid to fumaric acid. These results suggest that the increase of kinetic resistance is induced mainly by the higher acidity from succinic to fumaric acid and less by the increase of the acid—carrier compound structural complexity (in the same order). This differentiation becomes more important at lower concentration of sodium hydroxide in the stripping phase.

At higher pH_S_-value, implicitly at elevated concentration of the re-extraction agent, the magnitude of the effects of higher acidity or complexity of extracted compound structure is considerably attenuated. For pH_S_ > 10, the values of permeability factors of fumaric and malic acids exceed that of succinic acid (Fig. 5). This variation is the result of the more significant increase of the first two acids final mass flows related to that of succinic acid due both of the higher amounts of fumaric acid—Amberlite LA-2 and malic acid—Amberlite LA-2 compounds extracted in the liquid membrane and of the higher acidity of these two acids as compared to succinic acid.

**Figure 6:**
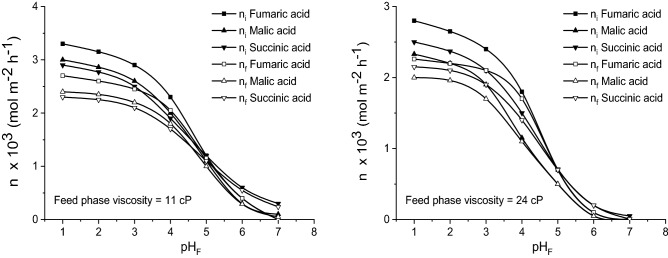
Influence of pH-value of feed phase on fumaric, malic, and succinic acids mass flows for viscous feed phase (carrier concentration = 200 g/l, pH of stripping phase = 10).

For more viscous feed phase, the general shape of the plotted dependences between the acids mass flows and pH_F_ remains similar to that for feed phase with 1 cP viscosity (Fig. 6). For all three dicarboxylic acids considered, the rate of transfer from feed phase to membrane one is reduced, due to the decreasing of their diffusion rates in aqueous phase towards the interface with the liquid membrane. Being dependent to the initial mass flows, the acids final mass flows are also diminished at higher viscosity.

However, by increasing the viscosity, from Fig. 6 it can be observed that the reduction of malic acid initial mass flow is more important. Therefore, for pH_F_ < 5 and feed phase viscosity of 11 cP, the values of this acid initial mass flow are closer to that of succinic acid, becoming lower that those at higher viscosity.

The increased magnitude of the relative influence of feed phase viscosity on the initial mass flow of malic acid is the result of this acid higher molecular weight compared to the other two acids, this amplifying the negative effect of the resistance to malic acid diffusion into the aqueous phase on the transfer towards the interface. The cumulated effects of the high viscosity and high molecular weight counteract the positive effect of higher acidity of malic acid compared to succinic one. As it was above discussed, the variations of the final mass flows are similar to the initial ones.

**Figure 7:**
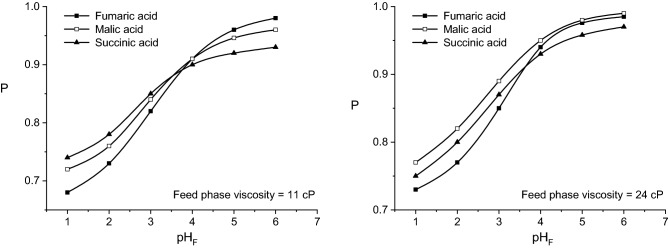
Influence of pH-value of feed phase on fumaric, malic, and succinic acids permeability factors for viscous feed phase (carrier concentration = 200 g/l, pH of stripping phase = 10).

Contrary to the influence on the acids mass flows, viscosity exhibits a positive influence on the permeability factors. According to Fig. 7, by varying the viscosity of feed phase from 1 to 24 cP, the permeability factor is increased by an average of 5–10%, the most important increased being recorded for lower pH_F_-values and malic acid. Obviously, this increase of the permeability factors is the consequence of the reduction of acids initial mass flows at higher viscosity, in the liquid membrane existing lower amounts of acid—carrier compounds, phenomenon more accentuated in the case of malic acid. For this reason, at 24 cP, the values of permeability factor corresponding to malic acid exceed the values related for the other two dicarboxylic acids (Fig. 7).

However, this increase of the membrane permeability cannot be correlated to an improvement of pertraction efficiency, because the transferred mass flows from the feed to stripping phase are decreased at higher viscosity of feed phase. The increased values of permeability factors suggest strictly that almost the entire amounts of acids transferred from the feed phase to liquid membrane are finally transferred to the stripping phase.

Because the stripping phase is not changed as rheological properties, the influence of the pH-value of stripping phase on dicarboxylic acids mass flows pertracted from viscous feed phase remains similar to that corresponding to the pertraction from aqueous solution with viscosity of 1 cP. By its effect on the acids mass flows from feed phase to liquid membrane, the variation of feed phase viscosity contributes indirectly to the influence of pH_S_ on acids mass flows or permeability factors. Therefore, compared to pertraction from aqueous solution of 1 cP viscosity, the increase of the viscosity of feed phase reduces the acids initial and final mass flows and increases their permeability factors for each considered pH_S_-value.

The variation of amine concentration into the liquid membrane exhibits similar effects on the pertraction efficiencies of these carboxylic acids, but with different relative magnitude. According to the previous works on reactive extraction of fumaric, malic, and succinic acids with Amberlite LA-2, the different importance of carrier influence is the result of the different acids extraction mechanisms, namely the number of aminic carrier included in the interfacial compound structure, as well as of the different acidity and hydrophobicity of the solutes^9,10^.

**Figure 8:**
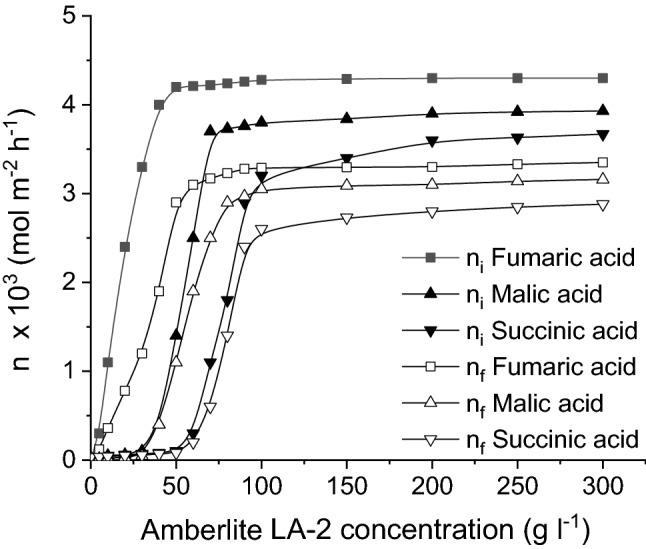
Influence of Amberlite LA-2 concentration on fumaric, malic, and succinic acids mass flows (pH of feed phase = 2, pH of stripping phase = 10, feed phase viscosity = 1 cP).

As can be seen from Fig. 8, the increase of carrier concentration induces a positive influence on all three acids mass transfers, due to the increase of Amberlite LA2 concentration at the feed/membrane interface that improves the reactive extraction performance generating the interfacial product buildup in the organic layer. Moreover, by increasing the carrier concentration into the liquid membrane, the following succession of the extraction of dicarboxylic acids from the feed phase was observed: fumaric acid, malic acid, and, finally, succinic acid. According to Fig. 8, for Amberlite LA-2 concentration below 30 g/l, fumaric acid represents almost the only acid transferred from the feed phase to the membrane one. The increase of carrier concentration allows to extract successively the other two acids too, namely malic acid for Amberlite LA-2 concentration over 30 g/l and succinic acid for Amberlite LA-2 concentration over 70 g/l. The observed phenomenon appears because the carrier reacts firstly with the solute possessing the highest acidity or with the solute that generates the simplest interfacial compound.

The mentioned values of Amberlite LA-2 concentration correspond to the stoichiometric need for the formation of the interfacial compounds of R(COOH)_2_.Q_2_ with fumaric and malic acids, or R(COOH)_2_.Q_4_ with succinic acid (8.6 × 10^−2^ M for reacting with fumaric acid, 7.4 × 10^−3^ M for reacting with malic acid, and 8.4 × 10^−3^ M for reacting with succinic acid, respectively). Practically, the pertraction of malic acid becomes possible for carrier concentration over 30 g/l, the initial mass flow of this acid being enhanced strongly for Amberlite LA-2 concentration varying from 30 to 70 g/l. Similarly, due to its lower acidity and more complex structure of the interfacially formed compound with the carrier, which includes four amine molecules, succinic acid is extracted from the feed phase only after the carrier concentration exceeds the sum of those stoichiometrically required for reacting with fumaric and malic acids (70 g/l). These results suggest that Amberlite LA-2 concentration could be an important factor controlling the pertraction selectivity.

The influence of the supplementary increase of carrier concentration over the mentioned stoichiometric limits exhibits an insignificant effect on initial mass flows of the three acids.

For carrier concentrations lower than those allowing transferring each acid from the feed phase to membrane one, the pertraction of weaker acids is possible only by physical solubilization in *n*-heptane. In this case, the initial mass flows of malic and succinic acids are superior to that of fumaric acid, because their dissociation degree at pH = 2 is lower.

Being in direct correlation with the amount of acids extracted into the membrane phase, the dependences between the acids final mass flows and the carrier concentration are similar to those above discussed for the initial mass flows.

**Figure 9:**
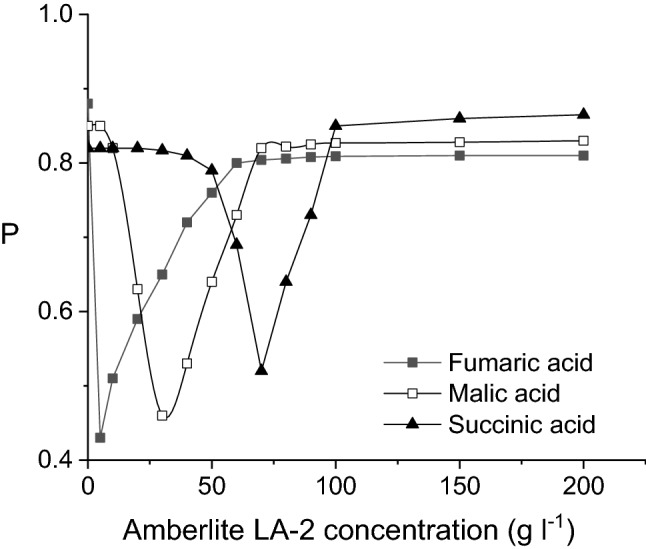
Influence of Amberlite LA-2 concentration on fumaric, malic, and succinic acids permeability factors (pH of feed phase = 2, pH of stripping phase = 10, feed phase viscosity = 1 cP).

The permeability factors through liquid membrane of the studied acids exhibit a particular variation with Amberlite LA2 concentration increase. According to Fig. 9, an initially decrease from 0 g/l aminic carrier into the liquid membrane (corresponding only to free pertraction) to a minimum value is registered, which depends on the pertracted acid. Therefore, the minimum value of permeability factor is reached at the concentration of 5 g/l Amberlite LA-2 for fumaric acid, 30 g/l for malic acid, while for succinic acid the minimum corresponds to 70 g/l carrier.

The recorded variations could be the explained by the modification of the relative rate of the chemical reactions occurring to the separation interface between the membrane and stripping phase. In the case of no Amberlite LA-2 in the liquid membrane, the extraction and transport of the acids through the organic solvent occur only by solubilization, controlled by diffusion, mechanism that is changed by carrier addition in *n*-heptane. The chemical reactions between the solute and the carrier at the feed phase—liquid membrane interface, as well as the chemical reactions between solute—carrier compounds and sodium hydroxide at the liquid membrane—stripping phase interface generates supplementary kinetic limitations. Furthermore, because at the re-extraction process the acids participate not in free acid form, but combined with the carrier, the rate of sodium salt formation is reduced. Therefore, the final mass flows will be initially smaller for the pertraction with Amberlite LA2 process as compared to the pertraction in which the membrane is formed only by *n*-heptane.

The decrease of the value of acidity index from fumaric acid to succinic acid improves the physical extraction from fumaric to succinic acid. Consequently, the amounts of free acids extracted in the membrane phase are higher for the malic and succinic acids. Due to this phenomenon cumulated with the superior acidity of fumaric acid, which amplified the kinetic resistance of the re-extraction process, the values of permeability factors recorded for malic and succinic acids are superior to that for fumaric acid, the highest values corresponding to the weakest acid, namely succinic.

**Figure 10:**
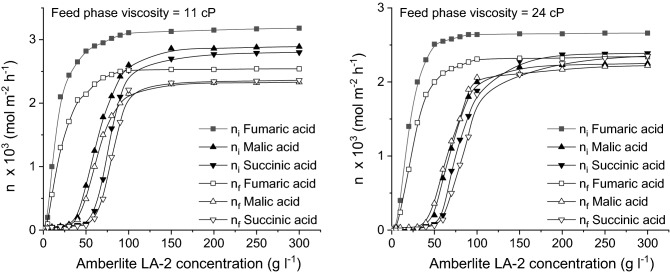
Influence of Amberlite LA-2 concentration on fumaric, malic, and succinic acids mass flows for viscous feed phase (pH of feed phase = 2, pH of stripping phase = 10).

By increasing the viscosity of feed phase, the relative magnitude of the carrier concentration influence is changed. As can be seen from Fig. 10, although the acids initial and, implicitly, final mass flows are reduced for higher viscosity, the general shape of the curves describing the dependence between the acids mass flows and Amberlite LA-2 concentration are similar to those recorded for the feed phase with 1 cP viscosity. Moreover, regardless of the viscosity value, by varying the carrier concentration from 5 to 300 g/l, the order of acids pertraction is maintained as in the case above discussed.

However, the most important negative effect of the viscosity increase is recorded for malic acid. For 11 cP, the initial and final mass flows of malic acids are closer to those of succinic acid, while for 24 cP become lower than the mass flows of succinic acid. This phenomenon is the result of the superior molecular mass of malic acid, which induces a higher resistance to the acid diffusion in the feed phase, effect amplified at higher viscosities. Therefore, the diffusion rate of malic acid through the feed phase towards the interface between this phase and the liquid membrane is lower than for the other two acids, its superior acidity compared to succinic acid being counteracted by its inferior diffusion rate.

The final mass flows are indirectly affected by the increase of feed phase viscosity, they depending on the initial mass flows. Consequently, the final mass flows of malic acid become closer or lower than those recorded for succinic acid (Fig. 10).

**Figure 11:**
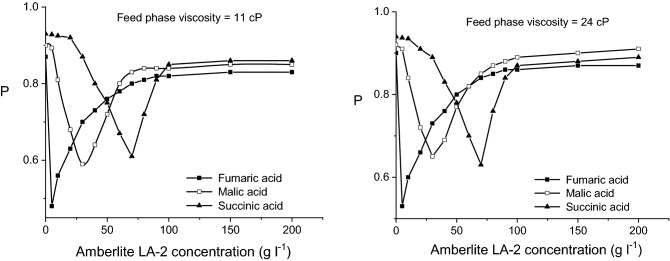
Influence of Amberlite LA-2 concentration on fumaric, malic, and succinic acids permeability factors for viscous feed phase (pH of feed phase = 2, pH of stripping phase = 10).

By analyzing the values of the permeability factors recorded for higher viscosities of feed phase, plotted in Fig. 11, two main aspects could be observed. On the one hand, the values of these factors are higher than those obtained for the viscosity of 1 cP, increasing for more viscous solutions. This variation can be attributed to the reduction of the three acids initial mass flows, due to the amplified diffusion resistance into the feed phase, the re-extraction process being not affected directly by this phase viscosity. On the other hand, for the above discussed reasons, the values of permeability factors of malic acid become closer to those of succinic acid and, finally, superior to those for both other two acids (Fig. 11).

These experimental data suggested that the mixture of these three studied dicarboxylic acids can be fractionated by facilitated pertraction with Amberlite LA-2. According to these results, fumaric and malic acids can be successively removed from the feed phase by pertraction, while succinic acid would remain in this phase.

In order to establish the validity of this assumption and the required conditions for reaching high selectivity of pertraction, the influences of pH-gradient between the aqueous phases and carrier concentration on pertraction have been studied in direct relation with the viscosity of feed phase. The selectivity of pertraction has been related to the main product of fermentation, namely fumaric acid, being described by means of the *selectivity factor*, S, defined as the ratio between the final mass flow of fumaric acid and the cumulated final mass flows of malic and succinic acids:

4 $${\text{S}} = \frac{{{\text{n}}_{{{\text{f}}.{\text{fumaric}}.{\text{acid}}}} }}{{{\text{n}}_{{{\text{f}}.{\text{malic}}.{\text{acid}}}} + {\text{n}}_{{{\text{f}}.{\text{succinic}}.{\text{acid}}}} }}.$$

**Figure 12:**
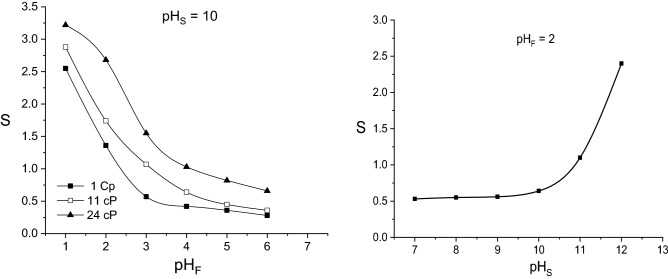
Influence of pH-values of feed and stripping phases on selectivity factor (carrier concentration = 200 g/l).

Figure 12 suggests that the increase of the pH-gradient between the feed and stripping phases leads to the increase of selectivity factor. Based on the above discussed effects of pH of the two aqueous phases on acids mass transfer, this variation is the result of the more important positive influence of lower pH_F_-values on extraction rate and of higher pH_S_-values on re-extraction rate of fumaric acid. Practically, the pertraction could be considered selective if S > 1, consequently for pH_S_ < 2.5 and pH_S_ > 10.

The negative effect of viscosity increase on the diffusion rate, mainly for malic acid, exhibits a positive influence on selectivity factor (Fig. 12). Because the magnitude of the reduction of initial and final mass flows of malic acid exceeds those of the reduction of the mass flows of the other two dicarboxylic acids, the ratio considered for calculating the selectivity factor is higher for more viscous feed phases. Therefore, the positive effect of lower pH_F_-values on factor S is amplified by increasing the viscosity of mixed acids initial solution.

For more viscous feed phases, the pH_S_-limit corresponding to S > 1 is moved to higher values (pH_S_ < 3 for 11 cP, respectively pH_S_ < 4 for 24 cP).

**Figure 13:**
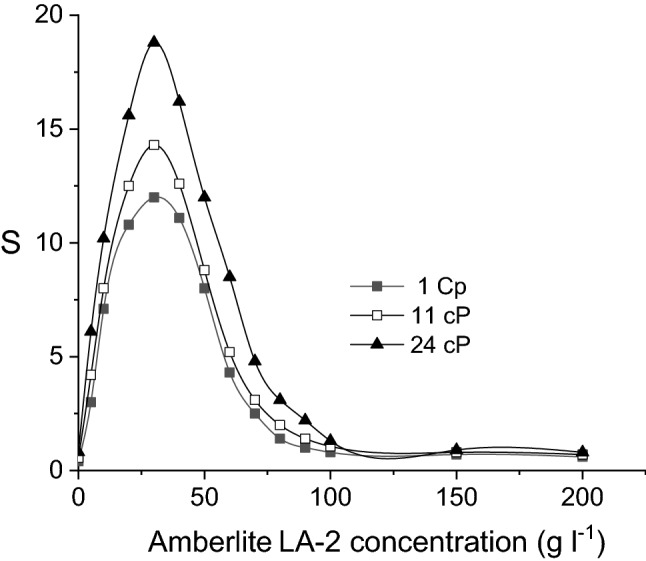
Influence of carrier concentration on selectivity factor (pH of feed phase = 2, pH of stripping phase = 10).

As it was discussed above, the influence of carrier concentration on pertraction selectivity is decisive, being underlined by the variation of selectivity factor plotted in Fig. 13. Therefore, for the entire domain of feed phase viscosity, the experimental data indicate that by increasing the Amberlite LA-2 concentration into the membrane phase the selectivity factor increases and reaches a maximum value, decreasing then. Regardless of the initial solution viscosity, the carrier concentration related to the maximum selectivity is 30 g/l, value which corresponds to the stoichiometry of the reaction with fumaric acid only. Moreover, the maximum values of selectivity factors are significantly higher than those recording by changing the pH- gradient between the two aqueous phases, in all cases S exceeding 10.

The increase of feed phase viscosity from 1 to 24 cP leads to the increase of maximum selectivity factor for 1.6 times, as the result of the resistance to diffusion in this phase, which is more important for malic acid.

Therefore, compared to the modification of the feed and stripping phase’s pH-values, the more important increase of the selectivity factor can be achieved by selecting properly the carrier concentration.

## Conclusions

The study on facilitated pertraction with Amberlite LA-2 dissolved in *n*-heptane of fumaric, malic, and succinic acids from their aqueous solution which simulates the composition and viscosity of the broth obtained by *Rhizopus oryzae* fermentation indicated that it is possible to separate selectively these dicarboxylic acids from their biosynthetic mixture. The results suggested that fumaric acid can be transferred from the feed phase through liquid membrane to the stripping phase, while malic and succinic acids remain in the feed phase.

The process parameter which exhibits the most important influence on selectivity is Amberlite LA-2 concentration into the liquid membrane. The influence of this parameter is related to the stoichiometry of the interfacial reaction between the aminic carrier and each dicarboxylic acid, the maximum selectivity factor reached by varying the carrier concentration being higher than 12. Consequently, the proper selection of Amberlite LA-2 concentration value cumulated with the appropriate pH-gradient between the aqueous phases allows recovering totally the main product of fermentation, fumaric acid, from the initial mixture.

By increasing the viscosity of the feed phase, the pertraction selectivity is improved, as the consequence of the amplification of solutes diffusion resistance, phenomenon which affects mainly the most voluminous acid, namely malic acid.

On the basis of these results, the aim of the future work is to verify these results for the real *Rhizopus oryzae* broths, because the presence of some cellular or biosynthetic compounds into the filtered or non-filtered broths (proteins, amino acids, etc.) could affect the pertraction efficiency and, implicitly, could lead to the change of some process conditions.

## Data Availability

All data generated or analyzed during this study are included in this published article.

## List of symbols

A: Interfacial area between the membrane phase and each of the two aqueous phases (m^2^)
C_0_: Acid concentration into the feed phase (mol/m^3^)
C_R_: Acid concentration into the raffinate (mol/m^3^)
C_S_: Acid concentration into the stripping phase (mol/m^3^)
n: Mass flow of carboxylic acid (mol/m^2^ h)
n_i_: Initial mass flow of carboxylic acid (mol/m^2^ h)
n_f_: Final (overall) mass flow of carboxylic acid (mol/m^2^ h)
P: Permeability factor (–)
S: Selectivity factor (–)
W: Volumetric flow rate (m^3^/h)
